# Metabolic Adaptation and Weight Regain in Obesity Treatment: The Central Role of Nutrition in the Era of Bariatric Surgery and GLP-1-Based Pharmacotherapy

**DOI:** 10.3390/nu18111725

**Published:** 2026-05-28

**Authors:** Larissa Vicente Pereira, Mário de Almeida Pereira Coutinho, Daniel Hortiz, Aline Lira Xavier, Raquel Patricia Ataide Lima

**Affiliations:** 1 Centro de Nutrição Clínica da Paraíba, João Pessoa 58032-090, PB, Brazil; larissa.viicente@gmail.com; 2 Hospital Universitário Lauro Wanderley, Federal University of Paraíba (UFPB), João Pessoa 58059-900, PB, Brazil

**Keywords:** obesity, metabolic adaptation, bariatric surgery, GLP-1 receptor agonists, clinical nutrition, weight loss maintenance, body composition

## Abstract

Obesity is a multifactorial chronic disease characterized by pathological adipose tissue expansion and systemic metabolic dysfunction. This review examines metabolic adaptation—the counter-regulatory physiological response to weight loss—and its contribution to weight recidivism. Although weight reduction confers substantial clinical benefit, long-term maintenance is frequently compromised by reductions in total daily energy expenditure (TDEE) that exceed predictions based on body composition. Bariatric surgery induces profound metabolic remodeling; however, its durability may be constrained by persistent biological defense mechanisms of body weight. Similarly, GLP-1 receptor agonists have transformed pharmacological management, yet in the absence of structured nutritional intervention, lean mass loss and energetic compensation may attenuate long-term stability. We propose an integrated model in which precision nutrition, surgery, and pharmacotherapy operate synergistically. Within this framework, nutrition should assume a central regulatory role in modulating body composition, substrate partitioning, and long-term energetic homeostasis to enhance the sustainability of clinical outcomes.

## 1. Introduction

Obesity is a chronic, multifactorial disease characterized by pathological adipose tissue expansion arising from the interaction between genetic susceptibility, neuroendocrine regulation, and environmental exposure [1,2,3,4]. Its escalating global prevalence has positioned it as a principal contributor to cardiometabolic disease, malignancy risk, systemic inflammation, and premature mortality [5,6].

Intentional weight reduction improves glycemic regulation, lipid metabolism, and inflammatory tone [7,8]. However, long-term weight stability remains the central therapeutic challenge. Across lifestyle, pharmacological, and surgical interventions, weight regain is common and reflects a biologically defended set-point rather than behavioral non-adherence alone [9,10].

Metabolic adaptation (adaptive thermogenesis) refers to the disproportionate suppression of energy expenditure relative to changes in body mass and composition following weight loss [10]. This response involves reductions in resting energy expenditure (REE), alterations in thyroid and sympathetic signaling, increased orexigenic drive, and attenuated satiety signaling [11]. These adaptations may persist beyond weight stabilization, contributing to biological pressure toward recidivism.

This manuscript is submitted as an Opinion article in accordance with the editorial scope of *Nutrients*. Rather than undertaking a systematic synthesis, it advances a physiologically integrated perspective on the interaction between metabolic adaptation, body composition remodeling, bariatric surgery, and incretin-based pharmacotherapy [12].

These domains are commonly addressed in isolation; however, their convergence within a shared framework of energy homeostasis, neuroendocrine signaling, and tissue-specific metabolic compensation remains insufficiently articulated [13].

Within this context, preservation of fat-free mass, particularly skeletal muscle, emerges as a central determinant of post-weight-loss metabolic homeostasis, given its influence on resting energy expenditure, inter-organ endocrine signaling, and functional metabolic capacity.

Recent reviews (2023–2024) have largely addressed metabolic adaptation, pharmacotherapy, and bariatric surgery as parallel but disconnected domains. Here, we take a different approach. Rather than examining these elements in isolation, this manuscript brings them together within a single, integrated framework of energy homeostasis and neuroendocrine regulation. In doing so, nutrition is repositioned—not merely as a behavioral tool—but as part of a broader, hierarchical metabolic system shaped by body composition, endocrine signals, and tissue-specific adaptations. Importantly, we also draw attention to a persistent translational gap: while the mechanisms underlying metabolic adaptation are increasingly well described, their application to long-term clinical practice remains limited.

Within this context, the preservation of fat-free mass emerges not as a secondary consideration, but as a central determinant of metabolic resilience. By advancing this integrative perspective, the present work seeks to move beyond reiterative synthesis and offer a more coherent physiological lens through which weight regulation can be understood.

This Opinion integrates evidence from lifestyle, pharmacological, and surgical interventions to examine the magnitude and durability of metabolic adaptation within the broader context of biological weight defense.

## 2. Metabolic Adaptation and the Role of Precision Nutrition

Metabolic adaptation refers to a disproportionate reduction in total daily energy expenditure (TDEE) and resting energy expenditure (REE) relative to changes in body mass and fat-free mass (FFM) following weight loss [12]. Evidence from controlled energy-restriction studies indicates that this suppression emerges early during caloric deficit and may persist after weight stabilization, although its magnitude varies considerably depending on methodological context and individual heterogeneity. This enhanced energetic efficiency increases vulnerability to positive energy balance and is considered a physiological component of weight recidivism [13,14].

Nevertheless, the quantitative contribution of adaptive thermogenesis to long-term weight regain remains variable and difficult to disentangle from behavioral compensation [15,16]. Reductions in leptin, insulin, and thyroid hormones signal central energy-conserving states in the reduced-weight condition [17,18]; however, whether these hormonal shifts act as primary drivers of relapse or represent proportional responses to diminished adiposity remains unresolved.

Beyond adipose tissue, skeletal muscle plays a critical role in this adaptive landscape. Alterations in muscle mass and quality directly influence REE, mitochondrial oxidative capacity, substrate utilization efficiency, and myokine-mediated endocrine signaling. Loss of skeletal muscle during weight-loss interventions may therefore exacerbate energy suppression and reduce functional capacity for physical activity, amplifying metabolic vulnerability in the reduced-weight state [19].

Although the biological robustness of metabolic adaptation is widely acknowledged, evidence that isolated nutritional strategies can durably override this response remains limited. Dietary approaches emphasizing higher protein-to-energy ratios, controlled energy density, and strategic meal distribution have been associated with enhanced satiety and relative preservation of lean mass [20,21]. These effects may indirectly modulate the post-weight-loss metabolic milieu; however, they do not constitute definitive evidence of sustained modification of the biologically defended body weight [22].

Interventions targeting lean mass retention, optimized protein intake, and postprandial glycemic regulation demonstrate strong physiological plausibility. Yet long-term randomized trials isolating these determinants as independent modulators of adaptive thermogenesis remain scarce [23]. Recent prospective data suggest that higher protein-to-energy ratios are associated with improved weight maintenance over 12 months, particularly when discretionary energy-dense foods are minimized [24]. Such findings support mechanistic coherence but warrant cautious interpretation regarding causal modification of metabolic set-point regulation.

Within this framework, precision nutrition is not positioned as a mechanism capable of fully suppressing metabolic adaptation, but rather as a potential modulator of the interaction between body composition dynamics, endocrine signaling, and long-term energetic stability.

## 3. Bariatric Surgery in Obesity Management: Metabolic and Nutritional Implications

Bariatric surgery remains the most effective intervention for severe obesity, particularly when lifestyle and pharmacological strategies fail to achieve adequate metabolic control [24]. Mean total body weight loss typically ranges from 20 to 35%, depending on procedure type and follow-up duration. However, long-term weight stability is not universal, and partial weight regain may occur in a subset of patients over time.

The metabolic effects of bariatric surgery extend well beyond mechanical restriction. Procedures such as Roux-en-Y gastric bypass (RYGB) and sleeve gastrectomy (SG) induce substantial reconfiguration of the gut–brain axis, including amplified incretin signaling, altered bile acid circulation, and modulation of central satiety pathways [25]. These neuroendocrine shifts contribute to improved glycemic regulation and appetite suppression, distinguishing surgical physiology from purely dietary weight loss.

Nevertheless, metabolic adaptation is not abolished postoperatively. Reductions in energy expenditure proportional to weight loss remain detectable, although the relative magnitude of adaptive suppression may differ from non-surgical interventions. Long-term weight durability appears to reflect the interaction between reduced adiposity, persistent neuroendocrine signaling, and changes in body composition [26,27,28].

RYGB and SG—the two most prevalent procedures globally—differ mechanistically and nutritionally. RYGB integrates gastric restriction with partial intestinal bypass, producing greater alterations in nutrient absorption and enteroendocrine signaling. SG operates predominantly through volumetric restriction and hormonal modulation, with comparatively lower malabsorptive impact [29]. These distinctions influence weight loss trajectories, metabolic outcomes, and nutritional risk profiles [30,31].

Observational cohorts and clinical trials consistently demonstrate reductions in all-cause and cardiovascular mortality, along with high initial rates of type 2 diabetes remission [32,33]. However, between two and five years post-procedure, some patients experience weight plateaus or regain—a multifactorial phenomenon involving both physiological compensation and behavioral adaptation [34].

From a body composition perspective, bariatric surgery is associated with substantial reductions in fat mass but also variable losses of fat-free mass. The early postoperative decline in energy and protein intake, combined with anatomical restructuring of the gastrointestinal tract, increases vulnerability to lean mass loss and micronutrient deficiencies without structured nutritional surveillance [35,36]. Preservation of skeletal muscle is therefore central not only to functional capacity and quality of life, but also to maintenance of resting energy expenditure and long-term metabolic stability.

Finally, although bariatric surgery provides robust efficacy, its scalability is constrained by eligibility criteria, procedural costs, and the requirement for lifelong follow-up. Thus, even within the surgical paradigm, structured and individualized nutritional management remains fundamental to sustaining metabolic durability and minimizing physiological vulnerability in the reduced-weight state.

Current clinical guidelines mandate high protein intake following bariatric surgery, typically ranging from 1.0 to 1.5 g/kg/day, to preserve fat-free mass (FFM) during the phase of rapid weight loss [37]. Furthermore, systematic micronutrient supplementation—including iron, vitamin B12, calcium, vitamin D, and folate—is considered obligatory, particularly following procedures with a malabsorptive component [38,39]. Recent evidence suggests that structured nutritional interventions focusing on dietary quality, protein adequacy, and metabolic monitoring are associated with superior weight maintenance and reduced recidivism risk post-surgery [40,41]. Thus, nutrition must not be viewed merely as postoperative support, but as a core therapeutic component for long-term surgical success [42].

The advent of glucagon-like peptide-1 (GLP-1) receptor agonists and dual GIP/GLP-1 agonists, such as semaglutide and tirzepatide, represents a paradigm shift in the pharmacological management of obesity and type 2 diabetes [43]. These agents are primarily indicated for individuals with obesity or overweight complicated by metabolic comorbidities—including insulin resistance, type 2 diabetes, and cardiovascular disease—especially when behavioral interventions alone prove insufficient [44]. Semaglutide operates predominantly by suppressing appetite, delaying gastric emptying, and enhancing central satiety, leading to a profound reduction in ad libitum energy intake [45]. Tirzepatide, by integrating both GLP-1 and GIP agonism, elicits more pronounced effects on glycemic control and weight reduction, often surpassing the outcomes observed with standalone GLP-1 agonists [46].

Recent clinical trials demonstrate that these therapies can induce substantial weight loss, frequently exceeding 10–20% of initial body weight [47,48]. However, mirroring the effects of hypocaloric diets and bariatric surgery, drug-induced weight loss is associated with significant declines in both total and resting energy expenditure, indicative of metabolic adaptation [49]. Emerging data suggest that the weight reduction promoted by semaglutide and tirzepatide is accompanied by a disproportionate loss of lean mass in the absence of targeted nutritional strategies. This loss can exacerbate the decline in energy expenditure and facilitate weight regain following treatment cessation [50,51]. Body composition studies reveal that without structured nutritional intervention, a clinically significant portion of GLP-1-induced weight loss may occur at the expense of fat-free mass [52].

The metabolic adaptation observed during pharmacotherapy appears primarily linked to sustained caloric restriction, which lowers basal metabolic requirements, alongside secondary hormonais shifts [53]. These mechanisms reinforce the concept that pharmacotherapy does not eliminate the biological defense of body weight but merely modulates it temporarily [54]. Consequently, nutrition plays a pivotal role in mitigating the metabolic adaptation associated with GLP-1 and GIP/GLP-1 agonists. Recent consensus recommends that patients receiving these therapies undergo nutritional counseling focused on protein adequacy (1.2–1.6 g/kg/day), lean mass preservation, and the maintenance of metabolic flux [55,56].

Furthermore, dietary strategies emphasizing micronutrient density, adequate fiber intake, and reduced energy density are recommended to sustain weight loss while mitigating the risk of nutritional inadequacies during prolonged pharmacotherapy [57]. Converging evidence indicates that treatment discontinuation in the absence of structured nutritional and behavioral support is frequently followed by substantial weight regain, reinforcing the biological resilience of body weight regulation [58,59].

Accordingly, nutritional management should not be conceptualized as ancillary to incretin-based pharmacotherapy, but rather as a central regulatory axis within the therapeutic framework. The coordinated integration of precision dietary planning, behavioral intervention, and resistance-based exercise may optimize body composition trajectories, preserve fat-free mass, and attenuate compensatory reductions in energy expenditure, thereby enhancing the durability of clinical responses to semaglutide and tirzepatide [60].

## 4. Discussion: Surgery, Pharmacotherapy, and Metabolic Adaptation in Obesity

Obesity is currently recognized as a chronic, relapsing, and biologically defended disease (Table 1). In this pathology, homeostatic and hedonic mechanisms operate in an integrated manner to preserve elevated body weight, persisting even in the face of effective therapeutic interventions [61,62]. Within this framework, both bariatric surgery and modern pharmacotherapy achieve substantial weight loss; however, both modalities interact with the same physiological systems that regulate energy balance, including metabolic adaptations that may undermine long-term weight maintenance [63].

Bariatric surgery remains the most efficacious intervention for inducing sustained weight loss in individuals with severe obesity. It functions not merely through mechanical restriction of caloric intake, but by triggering profound hormonal and metabolic shifts [64,65]. Procedures such as Roux-en-Y Gastric Bypass (RYGB) and Sleeve Gastrectomy (SG) are associated with durable changes in incretin secretion, insulin sensitivity, and central satiety signaling, all of which contribute to the magnitude of the observed weight loss [66,67].

Nevertheless, despite their robust short-term efficacy, longitudinal data indicate that a subset of patients develops weight-loss plateaus and gradual weight regain over time. These findings suggest that physiological defense mechanisms of body weight remain operative, even under potent therapeutic intervention, and may constrain the durability of treatment effects [68].

Metabolic adaptation following bariatric surgery is characterized by a reduction in energy expenditure that exceeds the decline predicted by changes in body mass and lean mass—a phenomenon consistent with the concept of adaptive thermogenesis [69,70]. Recent evidence indicates that this suppression of energy expenditure can persist for years post-surgery, particularly in individuals with significant loss of fat-free mass (FFM) or inadequate postoperative nutritional support [71]. These findings reinforce the premise that even after surgical intervention, the organism maintains physiological counter-responses aimed at restoring lost weight [72].

In parallel, obesity pharmacotherapy has advanced significantly, specifically with the development of glucagon-like peptide-1 (GLP-1) receptor agonists and dual GLP-1/GIP agonists. These agents are capable of inducing weight loss of a magnitude previously observed almost exclusively following surgical interventions [73,74].

These pharmacological agents exert their effects predominantly by reducing energy intake through the integrated modulation of hypothalamic circuits responsible for energy homeostasis and mesolimbic pathways—the dopaminergic neural systems involved in reward, motivation, and palatable pleasure—associated with hedonic appetite and food reward [75].

Emerging metabolic evidence indicates that pharmacotherapy-induced weight loss—paralleling observations in bariatric surgery—is accompanied by reductions in total energy expenditure that are not fully accounted for by changes in body composition alone. To date, consistent evidence demonstrating complete suppression of adaptive thermogenic mechanisms by these agents remains lacking, suggesting that biological weight-defense processes may persist, at least in part, despite highly effective pharmacological therapy [76].

Physiological evidence indicates that the decline in energy expenditure during semaglutide or tirzepatide treatment occurs proportionally to weight loss and alterations in body composition, further suggesting that central weight-defense mechanisms remain operative [77,78]. Moreover, the cessation of pharmacological treatment is associated with a rapid increase in appetite and weight recovery, a phenomenon compatible with the partial reversibility of pharmacological suppression over these systems [79]. These data support the notion that pharmacotherapy, in isolation, does not redefine the biological “set-point” of body weight [80].

In this scenario, nutrition emerges as a pivotal element in modulating metabolic adaptation for both bariatric surgery patients and those utilizing anti-obesity medications [81]. Severe energy restriction, common to both approaches, can compromise protein and essential micronutrient intake, favoring the loss of lean mass and amplifying the reduction in resting energy expenditure [82]. Recent evidence indicates that nutritional strategies with higher protein density, adequate diurnal distribution, and association with resistance training attenuate the magnitude of metabolic adaptation and enhance weight loss maintenance [83,84].

Furthermore, individualized nutritional approaches—considering body composition, treatment stage, and the specific intervention modality (surgical or pharmacological)—demonstrate superior efficacy in preserving lean mass and sustaining long-term negative energy balance [85]. Contemporary guidelines reinforce that the integration of surgery, pharmacotherapy, and nutrition should not be sequential but simultaneous and continuous, recognizing obesity as a condition requiring chronic management [86].

Guarnieri et al. (2025) [87] underscore that adequate protein and fiber intake, coupled with physical activity, can enhance satiety, energy expenditure, and lean mass preservation during weight loss—factors that favor weight maintenance. Similarly, Kokura (2024) [88] indicated that higher protein intake during weight loss can mitigate lean mass loss in adults with overweight or obesity, with clinical benefits observed when protein intake exceeds approximately 1.3 g/kg/day.

Taken together, the recent literature suggests that while both bariatric surgery and modern pharmacotherapy are potent tools for inducing weight loss, they do not eliminate the underlying physiological mechanisms of metabolic adaptation [89]. Clinical nutrition, coupled with structured physical exercise and longitudinal monitoring, remains the primary modulator of these processes and is the decisive factor for sustained weight maintenance and the prevention of weight recidivism [90]. Future research must focus on integrated strategies that aim not only for weight reduction but for a sustainable redefinition of energy balance and body composition in individuals with obesity [91].

## 5. Clinical Implications and Future Directions

Recognition of obesity as a chronic, biologically defended disease necessitates longitudinal therapeutic frameworks that extend beyond the magnitude of initial weight loss. While bariatric surgery and incretin-based pharmacotherapies (GLP-1/GIP receptor agonists) induce substantial reductions in body weight, neither intervention fully abrogates adaptive reductions in energy expenditure. Consequently, long-term efficacy should be evaluated in terms of metabolic durability, preservation of fat-free mass, and resistance to biological weight-defense mechanisms rather than short-term weight change alone.

Within clinical practice, bariatric surgery represents a potent metabolic intervention rather than a definitive therapeutic endpoint. Durable outcomes depend on structured nutritional management designed to limit lean mass loss, prevent micronutrient deficiencies, and preserve resting energy expenditure. Incretin-based therapies, by contrast, primarily induce weight loss through appetite suppression and delayed gastric emptying and do not directly target skeletal muscle preservation. Consequently, integration with resistance training and protein-optimized dietary strategies becomes critical to attenuate reductions in fat-free mass and functional capacity during pharmacologically induced weight loss.

In this framework, clinical nutrition assumes a regulatory rather than solely restrictive function. Beyond the induction of caloric deficit, dietary architecture modulates protein turnover, postprandial glycemic flux, substrate partitioning, and satiety-related neuroendocrine signaling. Strategies prioritizing optimized protein distribution, controlled energy density, and individualized adaptation may support metabolic stability in the reduced-weight state; however, long-term randomized trials isolating these determinants as independent modulators of adaptive thermogenesis remain scarce.

Pharmacotherapy discontinuation is frequently associated with weight regain, raising important considerations regarding treatment duration, cost-effectiveness, and equitable access. Given the economic constraints associated with lifelong pharmacological therapy and the procedural limitations of surgery, scalable nutritional frameworks remain indispensable components of comprehensive obesity management.

Future investigations should prioritize integrated models evaluating the interaction between surgery, pharmacotherapy, structured nutrition, and exercise on body composition dynamics and energy expenditure. Moving beyond total body weight as the sole outcome, research should address modulation of fat-free mass, neuroendocrine signaling, and metabolic flexibility. Such approaches may clarify whether long-term modification of biological weight defense is achievable or whether obesity management will continue to require sustained multimodal intervention analogous to other chronic diseases.

## Figures and Tables

**Table 1:** Comparative Physiological and Nutritional Dimensions of Major Obesity Interventions.

Intervention	Primary Mechanistic Driver	Dominant Metabolic Constraint	Body Composition Consideration	Strategic Role of Nutrition
Lifestyle Intervention (Caloric Restriction + Physical Activity)	Energy deficit-induced adipose tissue reduction with behavioral modulation	Adaptive thermogenesis and compensatory appetite signaling	Variable loss of fat-free mass depending on protein adequacy and resistance exercise	Optimization of protein distribution, energy density control, and preservation of lean mass to attenuate metabolic suppression
Pharmacotherapy (GLP-1/GIP receptor agonists)	Central appetite suppression and delayed gastric emptying with enhanced incretin signaling	Lean mass reduction and energy expenditure decline during rapid weight loss	Potential reduction in skeletal muscle mass without concurrent resistance training	Protein-optimized dietary structure and exercise integration to preserve fat-free mass and functional capacity
Bariatric Surgery (RYGB, SG)	Gut–brain axis reconfiguration, enhanced incretin response, altered bile acid signaling	Persistent adaptive reduction in energy expenditure; micronutrient vulnerability	Significant fat mass loss with variable fat-free mass reduction	Structured protein repletion, micronutrient supplementation, and long-term dietary surveillance to sustain metabolic stability

Reference: Compiled by the author.

## Data Availability

The data presented in this study are available on request from the corresponding author.
